# An In Vitro Evaluation of the Effect of Ceramic Material, Surface Treatment, and Adhesive Cement on Shear Bond Strength in Prosthodontics

**DOI:** 10.3390/medicina61071118

**Published:** 2025-06-20

**Authors:** Izabella Éva Mureșan, Diana Cerghizan, Attila Gergely, Rudolf-László Farmos, László Jakab-Farkas, John-Jason Șimon, Bernadette Kerekes-Máthé, Dóra-Anna Giliga, Esztella Éva Kis, Kinga Mária Jánosi, Krisztina Mártha

**Affiliations:** 1 Faculty of Dental Medicine, George Emil Palade University of Medicine, Pharmacy, Science and Technology of Targu Mureș, 38 Gh. Marinescu Str., 540139 Targu Mures, Romania; izabella-eva.muresan@umfst.ro (I.É.M.); bernadette.kerekes@umfst.ro (B.K.-M.); esztella.kis@umfst.ro (E.É.K.); kinga.janosi@umfst.ro (K.M.J.); krisztina.martha@umfst.ro (K.M.); 2 Department of Mechanical Engineering, Faculty of Technical and Human Sciences, Sapientia Hungarian University of Transylvania, 2 Calea Sighișoarei Str., 540485 Targu Mures, Romania; farmos_rudolf@ms.sapientia.ro (R.-L.F.); jflaci@ms.sapientia.ro (L.J.-F.); 3 Independent Researcher, 410057 Oradea, Romania; johnjasonsimon@gmail.com; 4 County Emergency Clinical Hospital of Targu Mures, 540139 Targu Mures, Romania; giliga.dora@yahoo.com

**Keywords:** shear bond strength, ceramics, adhesive cement

## Abstract

*Background and Objectives*: Ensuring the optimal shear bond strength (SBS) is essential for the long-term success of prosthodontic restorations. Our in vitro study aimed to evaluate the SBS of three types of ceramics (feldspathic, alumina, and lithium disilicates) using three adhesive cements (Variolink Esthetic LC, Variolink Esthetic DC, and Maxcem Elite). *Materials and Methods*: Healthy molars were prepared, and ceramic blocks were cemented following universally accepted luting protocols. SBS tests were performed using a custom-made testing machine. A multiple linear regression model assessed the effects of ceramic type, surface treatment, and luting agent on SBS. *Results*: The regression model explained 61.3% of the variation in SBS values (R^2^ = 0.613); the adjusted R^2^ = 0.605 confirmed the model’s robustness. The global F-test was statistically significant (F = 78.96, *p* < 0.001). The total-etch technique (+4.47), the use of feldspathic ceramic, and 5% hydrofluoric acid treatment (+3.28) significantly affected SBS. Feldspathic ceramic and lithium disilicate showed superior performance against alumina. Light-cured and self-cured cement showed negative effects. *Conclusions*: Ceramic material and cement type have combined effects on SBS. Optimal results were obtained with the total-etch technique, feldspathic ceramic, 5% hydrofluoric acid treatment, and dual-cured cement.

## 1. Introduction

Patient demand for esthetics has significantly increased in recent years, which has led to the development of minimally invasive dentistry using ceramic restorations. The minimal preparation involved focuses on preserving the hard dental tissue, especially the enamel [1,2]. Newly developed ceramic systems with improved structural qualities can restore the desired esthetics and have the necessary mechanical resistance to ensure the long-term success of prosthetic works [3,4,5]. Such systems are made of materials such as feldspathic ceramics, lithium disilicate, and alumina [6,7]. Feldspathic ceramics combine a glass phase (55–65% potassium aluminum silicate, sodium aluminum silicate) with a crystalline (silica) phase [6]. Feldspathic ceramics are considered ideal for rendering esthetics specific to natural teeth, achievable due to the translucency given by their glassy structure. Compared with lithium disilicate ceramics, the flexural resistance usually ranges from 60 to 70 MPa, which makes the material much more sensitive to mechanical stress. Feldspar blocks for CAD-CAM technology have a larger flexural strength (154 MPa), so they can be used in very thin layers of even less than 0.5 mm. Achieving this thickness requires minimal tooth preparation, and this thickness is ideal for veneers, inlays, onlays, and anterior single-unit crowns [6,8]. Lithium disilicate ceramics are an esthetic alternative with better mechanical properties, which belong to particle-filled glass ceramics. These materials were introduced on the market in the 1990s under the name “IPS EMPRESS 2” (Ivoclar Vivadent, Schaan, Liechtenstein), and their characteristics include a flexural strength of 350 MPa, a biaxial flexural strength of 407+/−45 MPa, a heat extrusion temperature of 920 °C, and an elastic modulus of 103 GPa [9,10]. “IPS e max Press” was launched in 2001 (Ivoclar Vivadent). This material is an optically and mechanically enhanced lithium disilicate, with such enhancements made possible thanks to improvements in the manufacturing process that ensure the formation of much smaller and more evenly embedded crystals [11]. The development and implementation of CAD-CAM technology on a growing scale has also led to the emergence of pre-crystallized ceramic blocks with lower flexural strength (130 MPa), reaching maximum flexural strength values of 262 ± 88 MPa after milling and heat treating (840–850 °C for 10 min) [10,12,13,14,15]. These ceramic blocks are considered to be extremely versatile materials, with indications such as crowns, anterior veneers, posterior inlays, onlays, and overlays [12]. Alumina is a suitable ceramic material for esthetic restorations in the posterior area of the dental arches. Alumina, which has a polycrystalline ceramic composition built up of a fine-grain crystalline structure that provides strength and fracture resistance to the material, was first introduced in the 1950s. Because of the lack of glassy phases, it has limited translucency. Even if it presents a very high Rockwell hardness (HRA 80–90) and strength, its elastic modulus (380 GPa) leads to vulnerability and bulk fractures [16,17,18,19]. This material has been the subject of remarkable developments over the years, with improvements being made to its mechanical properties via the microstructural refinement of alumina and alumina composite materials [18,19,20,21,22,23,24]. It has also been used in orthopedic applications, alongside bioceramics [25], and in dentistry. It is suitable in all areas of the mouth, making it viable for constructing frameworks of full-veneer crowns, fixed prostheses, and orthodontic brackets [19,26,27].

The same trend of significant development has been observed for dental luting cements. Proper cementation of indirect prosthetic restorations prevents biofilm formation at the finish line and helps to avoid the appearance of marginal gaps, coloration, and decay, minimizing mechanical and biological complications [28,29,30]. Recently, light-curing and dual- and self-adhesive luting systems have also emerged, helping to provide long-lasting fixation of the restorations to the dental structures. The long-term cementation can be performed with water-based (zinc polycarboxylate, zinc phosphate, conventional glass-ionomer or resin-modified glass-ionomer cement) and resin-based (self-curing, light-curing, dual-curing, adhesive, self-adhesive cement) cement [30]. The appearance of resin-based cement was imposed by the increasing demand for minimally invasive indirect ceramic restorations [31], providing the highest bond to the tooth surface [32]. For the long-term success of fixed prosthodontic restorations, the correct choice of luting cement and proper ceramic surface treatment is mandatory [33,34]. The surface treatment of ceramic materials before adhesive cementation can be performed using mechanical (airborne particle abrasion, surface acid etching, and grinding with diamond rotary instruments), chemical (universal or ceramic primers), or chemo-mechanical methods [35]. The choice of an adequate surface treatment method is influenced by the chemical composition of the ceramic materials [36]. The proper surface treatment for glass–ceramics (feldspar-based, leucite-reinforced, lithium disilicate, zirconia-reinforced lithium silicate ceramics, and fluorapatite ceramics) is acid etching [37], while for aluminum trioxide, the tribochemical silica-coating treatment [38] should be used. Hydrofluoric acid is commonly used for acid etching [36]. It provides roughness and microretentions on the ceramic surface that can lead to a higher resin-ceramic bond strength [39,40]. Acidulated phosphate fluoride and ammonium hydrogen difluoride can also be used for this purpose [35]. According to the universally accepted luting protocols, the duration of hydrofluoric acid application must be correlated with the used ceramic type: 20 s for lithium disilicate, 30 s for zirconia-reinforced lithium disilicate, 60 s for leucite-reinforced or feldspar-based ceramics, and 120 s for fluorapatite ceramics [35]. The luting cement is moisture-sensitive. Proper handling, according to the manufacturer’s indications, and humidity control are mandatory for the successful clinical application [30].

In a clinical context where restorative treatments must meet both functional and aesthetic requirements, understanding the interaction between ceramic type, surface treatments, and adhesive cement becomes essential for the long-term success of indirect restorations. The wrong choice of these variables can compromise the integrity of the restoration, leading to decementation, marginal infiltration, or even ceramic fractures. Although numerous studies have investigated the behavior of different types of ceramics or adhesive cement separately, there is a lack of research that systematically compares bond strength according to combinations of ceramics, surface treatments, and types of cement used [32,37,39].

This study aims to analyze the shear bond strength (SBS) of different ceramics (feldspathic, lithium disilicate, and alumina) cemented to enamel with various adhesive luting cements (light-cure, dual-cure, and self-adhesive) under different surface treatment conditions. The null hypothesis was that there is no significant difference in SBS among different ceramics, depending on the cement used and the surface treatments.

## 2. Materials and Methods

### 2.1. Study Design

This in vitro study was conducted at the Faculty of Dental Medicine of the University of Medicine, Pharmacy, Science, and Technology “George Emil Palade” in Târgu Mures. It was conducted in accordance with the Declaration of Helsinki. Approval was received from the ethical committee of The George Emil Palade University of Medicine, Pharmacy, Science, and Technology of Târgu Mureș (3011/1 April 2024).

Extracted molars were prepared for this study. Ceramic blocks were cemented on the axial surface of the teeth with different luting agents using different surface preparation protocols for the enamel and the different ceramic materials. The SBS was analyzed using a custom-made testing machine. The results were recorded and analyzed.

### 2.2. Sample Size

Post hoc power analysis using the statsmodels library in Python was performed to assess the sample size used for multiple linear regression models with two-way interaction terms. The model included 16 predictors (main effects and second-order interactions) and was built based on a complete factorial design with 81 unique experimental combinations (three ceramic materials × three cement types × three tooth surface preparation methods × three ceramic surface treatments). The full factorial design included three types of ceramics, three types of adhesive cement, and three methods of tooth surface conditioning, resulting in 27 theoretically possible combinations. However, only 21 experimental groups were initially considered for testing. Subsequently, six of these combinations were excluded due to incompatibility with adhesive protocols or lack of clinical relevance. Similarly, light-curing and dual-curing cement require a conditioned surface and were not assigned to the no-treatment groups. Thus, the final analysis included 15 experimental groups, each reflecting exclusively clinically valid combinations and compatible with the cementation protocols used in practice.

Overall, 510 teeth were evenly distributed, including approximately 6 replicas per group, ensuring a robust estimate of within-group variability. The resulting coefficient of determination was R^2^ = 0.613, which corresponds to a large effect size (f^2^ = 1.58). Given a significance level of α = 0.05, the calculated post hoc statistical power was 98.3%, demonstrating that the sample size was statistically sufficient for detecting the observed effects.

### 2.3. Tooth Preparation for This Study

Freshly extracted molars, including those extracted for orthodontic or periodontal reasons, were collected and prepared for this study.

The inclusion criteria were as follows: sound clinical crown, accurate dimension of the axial surface for ceramic block cementation, and healthy enamel on the axial surface.

The exclusion criteria were as follows: frontal teeth, premolars, decayed teeth, fillings or prosthetic restorations, and hypoplasia of the axial surface.

**Figure 1:**
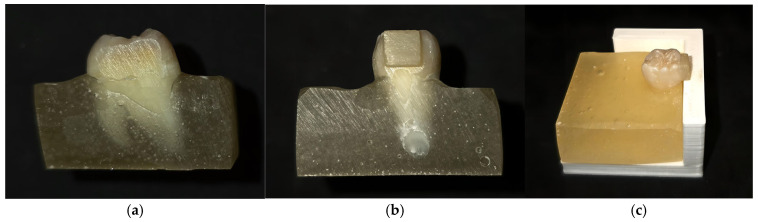
The samples prepared for the shear bond strength test: (**a**) the flat enamel surface obtained after using the Micracut 151 Metkon device (Metkon USA Inc. Greenville SC 29662, USA) at 500 rotations/min; (**b**) the luted ceramic block on the flat axial surface; and (**c**) the proper cementation of the ceramic block to the enamel surface, ensured by using a cementation guide.

Following tooth extraction, the teeth were debrided of residual plaque and calculus, sterilized using an autoclave (without drying), and kept in artificial saliva, which was changed weekly until the study was performed. While preparing the specimens, the teeth were embedded in self-polymerizing acrylate molds, leaving one of the axial surfaces uncovered by acrylic material. The Micracut 151 Metkon (Metkon USA Inc. Greenville SC 29662, USA) device was used at 500 rotations/min to obtain 5 mm^2^ flat surfaces on the axial surfaces of the teeth, allowing for the proper seating of the ceramic blocks with flat surfaces during the cementation process to minimize the amount of cement between the tooth surface and the ceramic blocks (Figure 1).

### 2.4. Preparation of Ceramic Blocks for This Study

Overall, 170 lithium disilicate blocks were obtained by CAD-CAM design and processing, and 170 feldspathic ceramic blocks were obtained by burning the ceramic layers in a silicon die, which allowed for the preparation of fragments of 5/5/2 mm size. For accurate burning, information from the manufacturer was used to calculate the material’s shrinkage so that, in the end, we could obtain fragments of 5/5/2 mm with no need for any dimensional adaptation.

The 170 alumina blocks were obtained by milling from ingots. Table 1 describes the chemical composition of the materials used in this study.

### 2.5. Cements Used in This Study

The resin cements used in this study (Variolink esthetic light-cure, Variolink dual-cure, and Maxcem elit dual-cure) were selected because they are frequently used in clinical practice for cementing minimally invasive restorations. Table 2 presents the cement’s composition and the materials used for the cementation process.

### 2.6. Tooth Preparation for Cementation

Regarding the cementation process, three key points were considered:

1. The total-etch technique was used. To condition the enamel surface, 37% orthophosphoric acid was used. The acid was left to act for 25 s, followed by thorough washing and drying of the surface. According to the manufacturer’s instructions, the bonding material (Adhese Universal—Ivoclar Vivadent) was applied to the enamel surface, and after proper surface activation (60 s), it was light-cured for 15 s.
2. The self-etch technique was also used, involving the use of a primer on the enamel surface for 20 s, followed by air drying. The bond was applied and activated on the enamel surface for 15 s, followed by light-curing for 10 s (OptiBond eXTRa Universal Adhesive, Kerr Corporation, Kloten, Switzerland).
3. Treating the enamel surface for cementing with self-adhesive cement (self-adhesion) is unnecessary.

### 2.7. Preparation of Ceramic Blocks for Cementation

The ceramic blocks were prepared for cementation according to the following universally accepted procedures:

1. The preparation of the feldspathic ceramic blocks involved acid etching with 5% hydrofluoric acid (HF, IPS ceramic etching gel) for 60 s [35], followed by proper washing and drying of the surface. An extraoral cleaning agent (Ivoclean, Ivoclar, Schaan, Liechtenstein) for indirect restorations was used for 60 s to remove intermediate compounds, followed by washing and drying of the surface. The primer (Monobond Plus, Ivoclar, Schaan, Liechtenstein) was applied for 60 s (continuously activated with the help of a disposable micro brush), followed by easy air drying.
2. For the lithium disilicate blocks, the luting protocol used was similar to that used for the feldspathic ceramic blocks. The key difference lies in the time required to ensure the proper action of hydrofluoric acid (5%), usually 20 s [35].
3. In terms of alumina, its absence of a glassy phase renders it difficult to etch with hydrofluoric acid, so the surfaces were prepared for luting by sandblasting with aluminum oxide particles (110 μm) under 2 bar pressure for 10 s [41].

All conditioning protocols applied to the dental and ceramic surfaces were selected following the manufacturers’ instructions for each adhesive system used. No cross-referencing between protocols specific to different cements was performed. Each experimental group reflected a clinically validated and commonly used combination, aiming to reproduce real-world cementation scenarios in dental practice faithfully.

Technical errors can occur during these multi-step procedures, influencing the mechanical properties and the resin–ceramic bond strength [42,43]. Ivoclar Vivadent has developed the first self-etching ceramic primer, simplifying the bonding process of glass ceramics and making it safer and less technique-sensitive [44,45]. To compare the effect of this self-etching ceramic primer and hydrofluoric acid–silane coupling surface treatments in terms of bond strength, the ceramic specimens (lithium disilicate and feldspathic ceramics) were divided into two groups: G1, representing specimens subjected to conventional surface treatment (HF and silane), and G2 representing specimens subjected to treatment using the self-etching ceramic primer (Monobond Etch &Prime, Ivoclar, Schaan, Liechtenstein). The self-etching ceramic primer was continuously activated on the ceramic surface with a micro brush for 20 s before being left for 40 s to ensure proper action. Subsequently, the ceramic sample was washed and air-dried.

Thus, feldspathic ceramic, lithium disilicate ceramic, and alumina blocks were luted by light-cured (Variolink LC, Ivoclar, Schaan, Liechtenstein) and dual-cured (Variolink DC) cement to the conditioned enamel or by using a dual-cure and self-adhesive cement (Maxcem Elite, Kerr Corporation, Kloten, Switzerland) without surface conditioning. The cementation protocols used are presented in Table 3.

After the teeth and ceramic blocks were subjected to surface conditioning, the resin cements were prepared according to the manufacturer’s instructions. The ceramic blocks were maintained under digital pressure throughout the total setting of the resin cement [46]. A Coltene SPEC 3 light-curing lamp (Coltene/Whaledent AG, Canton St. Gallen, Switzerland) was used to photopolymerize the cement until the complete setting of the material. This lamp features a 430–490 nm wavelength range with a 1600 MW/cm^2^ ± 10% irradiation intensity in the standard mode. All these technical data were used to choose the proper light-curing time for the materials included in this study, taking into consideration the recommendations of the manufacturers.

### 2.8. Shear Bond Strength (SBS) Testing

A custom-made testing machine equipped with a calibrated 500 N force gauge (Axis FB 500 N, Axis, Gdansk, Poland) was used to perform the SBS tests. The specimens were fixed to the platform of the testing machine using clamps. Shear loading was applied under quasistatic conditions. The debonding force (F) for each specimen was recorded, and the shear force was calculated using equation SBS = F/A, where A is the cross-section area (in mm^2^); the value of SBS was determined in MPa. The results were averaged on 5 specimens. The SBS tests were carried out under standard laboratory conditions. After debonding, the tooth surfaces were analyzed for failure types with the help of a custom-made setup. Images were acquired using a Nikon Z8 mirrorless full-frame camera (Japan) equipped with a 35.9 × 23.9 mm sensor, offering an inherent resolution of 8256 × 5504 pixels. A Tamron SP AF 90 mm f/2.8 Di Macro lens (Japan) was used at a magnification of 0.95×. The images were originally recorded in RAW format and subsequently processed using Adobe Photoshop Lightroom Classic version 14.2. Final image dimensions were cropped to 2430 × 2430 pixels, yielding a spatial resolution of 220 pixels/mm. Bonding failure was categorized as cohesive (failure within the tooth structure or cement itself), adhesive (failure between the cement and tooth structure or between the cement and ceramic blocks), or mixed.

### 2.9. Statistical Analysis

Statistical analysis was performed using GraphPad Prism 8 for macOS (version 10.3.1 (464)) and Python (statsmodels, version 3.13.4), with the significance threshold set at *p* < 0.05. Descriptive statistics were calculated, and the Shapiro–Wilk test revealed significant deviations from normality across all experimental groups, warranting robust statistical methods.

A multifactorial ANOVA with fixed effects was applied to evaluate the individual contribution of the four experimental factors (ceramic material, cement type, surface preparation method, and ceramic treatment) to SBS.

A multiple linear regression model with interaction terms was constructed to model the joint effect of experimental conditions. This approach allows for the simultaneous estimation of main and interaction effects, improving explanatory power (R^2^ = 0.613) compared to additive models. Model validity was confirmed through the adjusted R^2^, AIC (Akaike information criterion), BIC (Bayesian information criterion), and RMSE (root mean square error), while robustness was assessed using the variance inflation factor (VIF), studentized residuals, and Cook’s distance. The chi-squared test was used to assess the association between the type of ceramic material and failure mode.

## 3. Results

**Figure 2:**
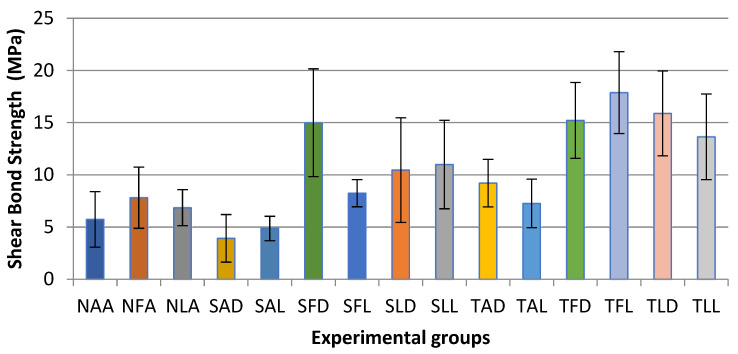
Distribution of SBS mean values by experimental group.

Descriptive statistics were calculated for each of the 15 experimental groups included in this study. Table 4 presents each group’s SBS mean values, standard deviations (SDs), and minimum and maximum values (Figure 2).

A multifactorial ANOVA with fixed effects was applied to evaluate the individual influence of each experimental factor on SBS values. This approach allowed us to distinguish the contribution of each main factor (ceramic material, type of cement, tooth surface preparation method, and ceramic treatment) and is appropriate for factorial experimental designs (Table 5).

The post hoc Tukey HSD test revealed statistically significant differences (*p* < 0.05) between experimental groups defined by the combination of surface treatment type, ceramic material, and cement type used. The most pronounced differences in SBS were observed between the TFL and NAA (Δ = +11.91 MPa, *p* < 0.001), SFD and NLA (Δ = +9.25 MPa, *p* < 0.001), and TLL and NFA (Δ = +9.36 MPa, *p* < 0.001) groups, respectively. These results indicate the systematic superiority of protocols that include active chemical treatments on the ceramic (total-etch or self-etch) in association with LC or DC cement compared to protocols without ceramic treatment and with self-adhesive cement. The TFL, TLL, and SFD groups recorded statistically significant and consistent differences compared to most untreated groups (such as NAA, NLA, and NFA). All significant differences identified had a positive sign, confirming an increase in SBS values in favor of the treated groups.

A multiple linear regression model with interaction terms was used to simultaneously estimate experimental variables’ main effects and interactions on SBS values. The model explained 61.3% of the variance in SBS values (R^2^ = 0.613), with an adjusted R^2^ of 0.605, indicating a satisfactory model fit (Table 6). Model quality was assessed using the AIC and BIC, and the RMSE was approximately 3.24 MPa. Multicollinearity was evaluated using the VIF, with values < 10, suggesting the absence of excessive collinearity.

The influence of individual observations was assessed using Cook’s distance and studentized residuals, with no extremely influential values being identified. The agreement between the observed and predicted values of shear strength supports the validity of the multiple regression model and strengthens the robustness of the statistical results.

The coefficients of the interaction terms Ceramic material × Cement type, Ceramic material × Etching, and Etching × Cement type were calculated to highlight the combined effects of experimental factors on SBS values. The results are presented in Table 7, Table 8 and Table 9.

**Figure 3:**
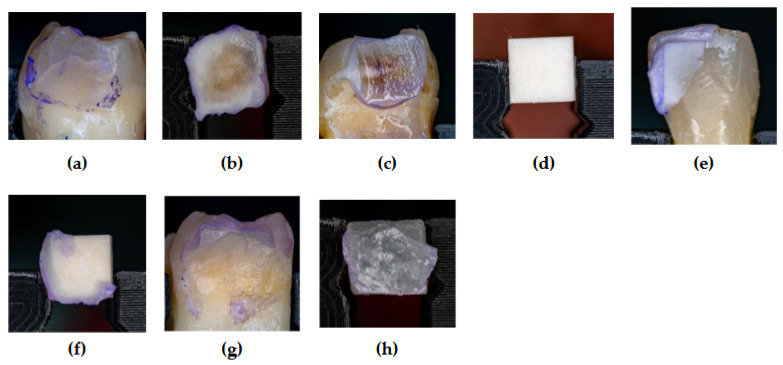
The aspect of the tooth and ceramic surface after SBS testing: (**a**) adhesive failure—lake of cementum on the enamel surface; (**b**) adhesive failure—luting cement (DC) on the ceramic fragment (felspathic); (**c**) adhesive failure—cementum fragments (DC) on the enamel; (**d**) adhesive failure—lake of cementum on the ceramic fragment (alumina); (**e**) cohesive failure—cementum fragments (DC) on the enamel; (**f**) cohesive failure—cementum fragments (DC) on the ceramic block (alumina); (**g**) mix failure—luting cement (LC) and ceramic fragments on the enamel; (**h**) mix failure—cementum fragments (LC) on the fractured ceramic (feldspathic).

The chi-squared test indicated a significant association between the type of ceramic material and failure mode: χ^2^ = 308.69, *p* < 0.0001. This suggests that the material type influences the nature of the observed failure, as represented in Figure 3.

## 4. Discussion

The interaction between tooth surface, ceramic treatment, and cement type significantly influences the SBS. HF is considered the gold standard in the surface treatment of glass ceramics [47]. It provides a reliable bond between the tooth and the restoration, essential in preventing marginal gaps, discoloration of the cemented restorations, and decay, minimizing mechanical and biological complications [28,29,30]. In this study, ceramic treatment with HF demonstrated a favorable effect (+3.28 MPa), which confirms its gold standard status for conditioning glass ceramics. In their study, Gupta et al. compared the efficiency of different ceramic (feldspathic) surface treatment techniques involving HF, coarse diamond burs, and a CO_2_ laser. Surface treatment with HF was proven to be the most efficient method, resulting in the highest bond strength [48]. In addition to the etching protocol, luting cements provide the bonding force to the dental tissue. Naumova et al. also demonstrated the importance of the tooth surface during adhesive cementation by revealing that ceramic blocks cemented to enamel have higher SBS values compared to dentin [49]. Considering that the enamel thickness decreases during aging [50], proper case evaluation and planning are essential for the long-term success of adhesive restorations. In this study, the ceramic blocks were luted to enamel. Numerous methods of analyzing bond strength can be found in the literature. Oilo investigated and compared the accuracy of various tests (tensile and micro-tensile tests, shear and micro-shear tests, and three-point bending tests). He found that the shear bond strength test is the most frequently used evaluation method [51], as we also found during our research. Self-adhesive resin cements are the most used in clinical practice due to their structural characteristics, simplifying the cementation protocols. However, Calheiros-Lobo highlights in her systematic review and meta-analysis that their performance is substrate-dependent [52]. Mondal et al. analyzed the SBS of lithium disilicate using one LC cement and two DC resin cements. Their study’s results highlight the efficiency of DC resin cement, which achieved the highest SBS values [53]. These results are in accordance with those of Alqahtani et al. [54]. Our results, similar to the above studies, showed that DC resin cement had the highest SBS values. We have also found that ceramic material directly influences adhesive performance. The feldspar and lithium disilicate ceramics demonstrated significant positive coefficients compared to alumina. Sandblasting the alumina resulted in a significant negative effect (–1.90 MPa), suggesting limited adhesive efficiency in the absence of a vitreous phase. The association of feldspar or lithium disilicate ceramic with self-adhesive cements showed a negative effect, with a decrease of up to –6.46 MPa, which indicates chemical incompatibility between these compounds.

Several experimental studies have compared the effect of self-etching ceramic primer, HF, and silane on the SBS of ceramic materials. The results are contradictory due to the significant variability of ceramic materials and testing methods. Vila-Nova et al. did not find any significant differences between the SBS of the LD when analyzing two different etching procedures [55]. In contrast, others have concluded that better results can be obtained with self-etching ceramic primers [45]. Awad et al. conducted a meta-analysis to examine the bond strength of dental glass ceramics treated with self-etching primer and HF etching gel. Their study demonstrated that self-etching primer leads to significantly lower bond strength than HF and silane application [56]. In this study, conventionally etched ceramic samples showed higher SBS values than those treated with self-etching primers. A study by Alsolami et al. proposed a new surface treatment protocol that involved increasing the application time of the self-etch primer (Monobond Etch & Prime) to two minutes to obtain a more durable adhesion [57]. Further investigations are needed, but Monobond Etch & Prime could serve as a potential alternative to HF, despite the more significant safety concerns, as it could have less harmful effects on the human body. Attempts have been made with phosphoric acid and fluoride-based products such as Monobond Etch & Prime, but they cannot match the performance of HF acid etching [58,59,60]. Nishizawa et al. studied a new etching material, a mixed aqueous solution of ammonium fluoride and ammonium hydrogen sulfate, which has proven to be a less toxic alternative to hydrofluoric acid. The SBS values of materials (feldspathic ceramic, lithium disilicate, polymer-infiltrated ceramic, resin composites) treated with the new etching solution were comparable to those treated with HF [61]. The influence of the adhesive system on the adhesion force of resin cement is a theme in the literature. The highest SBS values were recorded when resin cement and the total-etch system were combined, followed by self-etch and self-adhesive systems [62]. Zhang et al. confirmed that multi-step dual-cured luting resins exhibit higher bond strength values than single-step auto-adhesive resin cements [63].

The two main indications for alumina in dentistry are fixed restorations and orthodontic brackets. The bonding strength of alumina brackets is often analyzed in the literature. The total-etch system combined with composite (Microarch, GAC, Bohemia, NY bracket, Evicrol, SpofaDental, Jièin, Cehia composit) exhibits the highest tensile bond strength, highlighting the importance of proper surface treatment in achieving maximum values of bond strength [64]. Our results are in accordance with those reported in the literature: positive interactions were identified at Total-etch × LC cements, with an average increase in SBS of +2.21 MPa, highlighting the importance of an adhesive protocol in several steps to maximize adhesion. In addition, the significant differences identified by the Tukey HSD post hoc test between untreated groups (NAA, NLA, NFA) and chemically treated groups (TFL, TLL, SFD) confirm the superiority of the combined protocols that include chemical conditioning treatments and LC or dual cements. This is supported by both the positive regression coefficients and significantly higher mean SBS values in these groups (e.g., TFL = 17.88 MPa vs. NAA = 5.73 MPa). The TFL, TLL, and SFD groups recorded significant and consistent statistical differences from most untreated groups (such as NAA, NLA, and NFA). All significant differences identified were positive, confirming an increase in SBS values in favor of the treated groups, which supports the assumption of a positive contribution of chemical surface treatments and advanced types of cementing to the improvement in adhesion performance.

Several methods are described in the literature for the simulation of the aging process, including keeping the bonded specimens in water for a variable time and thermocycling [53]. According to Abdullah et al., in the case of Variolink II cement (cement underlying the development of Variolink light-cured), SBS values did not show statistically significant changes after the aging process (before: 19,396 MPa; after: 20,936 MPa), and the values of dual-cured luting cement were significantly lower after the aging process (before 23,549 MPa; after 15,435 MPa) [65]. The importance of the interface (in our case, the bonding layer) in cementation has been highlighted in numerous studies. The adhesive and cohesive debonding method is the most commonly described in the literature, often described in the context of self-adhesive resin cements [62,65,66]. This supports the notion that SBS is influenced by the chemical composition of the cement, in the case of self-adhesive cement, allowing only superficial interaction with the enamel and minimal infiltration at this level [65]. In our study, several failure modes (e.g., cohesive, mix) are negatively associated with SBS values, which is consistent with theoretical expectations that samples prone to these types of failure exhibit poorer adhesive performance. Although self-adhesive cement requires the fewest work stages, multiple errors that would negatively influence cementation quality are eliminated. The low recorded SBS values explain why it has had limited use in fixed prosthodontics. In addition, light-curing cement ensures the longest working time and the best color stability compared to dual-cured and self-adhesive resin cement [67,68].

Lithium disilicate should be cemented with a total-etch adhesive system to obtain maximal adhesion to the dental tissue [7,56].

The overall model assessment by indicators such as adjusted R^2^ (0.605), RMSE (~3.24 MPa), VIF (<10), and Cook’s distance confirmed the statistical robustness and absence of collinearity or extreme influences.

## 5. Conclusions

Although self-adhesive cementing features the fewest work stages, thus eliminating multiple errors that would negatively influence the quality of cementing, the low SBS values recorded support the limitation of its use in fixed dental prosthetics. Lithium disilicate should be luted with light-cured cement and a total-etch adhesive system to achieve maximal adhesion to the tooth structures. With higher SBS values and improved mechanical and optical qualities, lithium disilicate is a viable alternative to feldspathic ceramics in the frontal region of the dental arches. Among the studied ceramics, aluminum oxide showed the lowest SBS values.

## Figures and Tables

**Table 1:** Characteristics of the used ceramic blocks.

Material	Commercial Name	Manufacturer	Components
Feldspathic ceramic	Vita Mark II,	Vita Zanhfabrik, Bad Säckingen, Germany	SiO_2_—56.0–64%, Al_2_O_3_—20–23%, Na_2_O—6–9%, K_2_O—6–8%, CaO—0.3–0.6%, TiO_2_ < 0.1%
Lithium disilicate	IPS e. max Press	Ivoclar Vivadent, Schaan, Liechtenstein	SiO_2_—57–80%, Li_2_O—11–19%, K_2_O—0–13%, P_2_O—50–13%, ZrO_2_—0–8%, ZnO—0–8%, Al_2_O_3_—0–5%, MgO—0–5%, Coloring oxides—0–8%
Aluminum oxide	InCeram Alumina	Vita Zanhfabrik, Bad Säckingen, Germany	Al—65.2%, Si—6.1%, Ca—1%, La—27.6%

**Table 2:** Materials used for the cementation process.

Material	Commercial Name	Manufacturer	Components
Materials for tooth surface preparation			
Etching gel	Total Etch Refill	Ivoclar Vivadent, Schaan, Liechtenstein	37% phosphoric acid
Universal adhesive	OptiBond eXTRa Universal Adhesive	Kerr Corporation Kloten, Switzerland	Ethanol 20–40%, 2-hydroxyethyl methacrylate 10–20%, glycerol dimethacrylate 1–10%, glycerol phosphate dimethacrylate 1–10%, sodium hexafluorosilicate <5%
Primer	Monobond Plus	Ivoclar Vivadent, Schaan, Liechtenstein	Phosphoric acid methacrylate, silane methacrylate, sulfide methacrylate
Bonding	Adhese Universal Vivapen		2-hydroxyethyl methacrylate ethanol, Bis-GMA 10-decandiol dimethacrylate methacrylated phosphoric acid ester, 2-dimethylaminoethyl methacrylate
Materials for ceramic surface preparation			
Ceramic etching gel	IPS ceramic etching gel	Ivoclar Vivadent, Schaan, Liechtenstein	Hydrofluoric acid
	Monobond Etch&Prime		Hydrofluoric acid, silane
Cleaning solution	Ivoclean		Polyethylene glycol 2.5–10%, sodium hydroxide ≤2.5%
Light-cured cement	Variolink Esthetic LC		Ytterbium trifluoride 20- < 25% *w*/*w*, urethane dimethacrylate 5- < 10% *w*/*w*, glycerin-1.3-dimethacrylate 5- < 10 *w*/*w*, 1,10-decandiol dimethacrylate 3–7% *w*/*w*
Dual-cured cement	Variolink Esthetic DC		Ytterbium trifluoride 10- < 25%, urethane dimethacrylate3- < 10%, 1,10-decandiol dimethacrylate 3- < 10%, acetyl-2-thiourea 0.3- < 1%
Dual-cured/Self-adhesive cement	Maxcem Elite	Kerr	Barium aluminoborosilicate glass 30–60%, ytterbium fluoride 10–30%, 1,6-hexanediyl bismethacrylate 5–10%, 2-hydroxy-1,3-propanediyl bismethacrylate 5–10%, 7,7,9(or 7,9,9)-trimethyl-4,13-dioxo-3,14- dioxa-5,12-diazahexadecane-1,16-diyl bismethacrylate 1–5%, 3-trimethoxysilylpropyl methacrylate 1–5%, Fumed silica 1–5%

**Table 3:** The cementation protocols used, along with information regarding surface conditioning, cement type, and type of ceramic material.

Surface Etching	Ceramic Type	Cement Type	Abbreviation
Total-etch	feldspathic ceramic	light-cured	TFL
		dual-cured	TFD
	lithium disilicate	light-cured	TLL
		dual-cured	TLD
	alumina	light-cured	TAL
		dual-cured	TAD
Self-etch	feldspathic ceramic	light-cured	SFL
		dual-cured	SFD
	lithium disilicate	light-cured	SLL
		dual-cured	SLD
	alumina	light-cured	SAL
		dual-cured	SAD
No treatment	feldspathic ceramic	dual-cured and self-adhesive	NFA
	lithium disilicate		NLA
	alumina		NAA

**Table 4:** Descriptive statistics for experimental groups.

Group	N	Mean	SD	Min	Max
NAA	30	5.73	2.66	2.72	8.9
NFA	36	7.81	2.93	4.94	12.1
NLA	36	6.85	1.72	4.6	9.54
SAD	30	3.92	2.28	1.36	7.9
SAL	30	4.86	1.17	3.38	6.22
SFD	36	14.99	5.17	8.26	20.0
SFL	36	8.24	1.3	7.05	10.11
SLD	36	10.46	5.02	5.59	19.56
SLL	36	10.99	4.24	5.62	17.1
TAD	30	9.21	2.28	6.58	12.37
TAL	30	7.26	2.32	4.09	10.94
TFD	36	15.21	3.64	8.08	20.0
TFL	36	17.88	3.92	9.41	20.0
TLD	36	15.88	4.07	9.84	20.0
TLL	36	13.64	4.1	7.35	20.0

**Table 5:** Multifactorial ANOVA results.

Factor	Sum of Squares	df	F-Statistic	*p*-Value
Ceramic material	6159.31	2	244.84	5.59 × 10^−75^
Cement type	408.57	2	16.24	1.46 × 10^−7^
Tooth preparation	8486.61	2	337.35	1.20 × 10^−93^
Ceramic treatment	1872.18	2	74.42	4.87 × 10^−29^
Residual	6326.89	503	—	—

**Table 6:** Regression coefficients for main and interaction effects on SBS.

Term	Coefficient	Std. Error	t-Statistic	*p*-Value	CI 2.5%	CI 97.5%
Intercept	6.1441	0.183	33.623	<0.001	5.785	6.503
Lithium disilicate vs. alumina	3.0591	0.358	8.555	<0.001	2.357	3.762
Feldspathic ceramic vs. alumina	4.9908	0.358	13.957	<0.001	4.288	5.693
Self-adhesive vs. dual-cured cement	1.4944	0.543	2.752	0.006	0.428	2.561
Total-etch vs. no treatment	4.4737	0.281	15.903	<0.001	3.921	5.026
Hydrofluoric acid vs. no treatment	3.2799	0.358	9.152	<0.001	2.576	3.984
Sandblasting vs. no treatment	–1.9058	0.345	–5.518	<0.001	–2.584	–1.227
Lithium disilicate × light-cured cement	–0.3519	0.840	–0.419	0.676	–2.003	1.299
Feldspathic ceramic × light-cured cement	–1.5389	0.840	–1.831	0.068	–3.190	0.112
Lithium disilicate × self-adhesive cement	–5.4829	1.029	–5.327	<0.001	–7.505	–3.461
Feldspathic ceramic × self-adhesive cement	–6.4626	1.029	–6.278	<0.001	–8.485	–4.440

**Table 7:** Ceramic material × cement type interactions.

Interaction	Coefficient	Std. Error	t-Statistic	*p*-Value	CI 2.5%	CI 97.5%
Lithium disilicate × light-cured cement	–0.3519	0.840	–0.419	0.676	–2.003	1.299
Feldspathic ceramic × light-cured cement	–1.5389	0.840	–1.831	0.068	–3.190	0.112
Lithium disilicate × self-adhesive cement	–5.4829	1.029	–5.327	<0.001	–7.505	–3.461
Feldspathic ceramic × self-adhesive cement	–6.4626	1.029	–6.278	<0.001	–8.485	–4.440

**Table 8:** Ceramic material × etching interactions.

Interaction	Coefficient	Std. Error	t-Statistic	*p*-Value	CI 2.5%	CI 97.5%
Lithium disilicate × total-etch (TLD)	+2.87	0.94	+3.06	0.002	+1.03	+4.71
Feldspathic ceramic × total-etch (TFD)	+2.45	0.91	+2.69	0.007	+0.66	+4.24
Lithium disilicate × self-etch (SLD)	+1.89	0.96	+1.97	0.050	+0.01	+3.77
Feldspathic ceramic × self-etch (SFD)	+1.54	0.94	+1.64	0.105	–0.32	+3.40

**Table 9:** Etching × cement type interaction.

Interaction	Coefficient	Std. Error	t-Statistic	*p*-Value	CI 2.5%	CI 97.5%
Total-etch × light-cured cement	+2.21	0.83	+2.66	0.008	+0.59	+3.84
Total-etch × dual-cured cement	+1.89	0.85	+2.21	0.027	+0.22	+3.57
Self-etch × light-cured cement	+1.35	0.81	+1.67	0.096	–0.24	+2.94
Self-etch × dual-cured cement	+0.89	0.84	+1.06	0.291	–0.75	+2.54

## Data Availability

The dataset analyzed during this study is available from the first author upon request.

## Abbreviations

The following abbreviations are used in this manuscript:

SBS	Shear bond strength
LC	Light-cured
DC	Dual-cured
CAD-CAM	Computer-aided design–computer-aided manufacturing
HF	Hydrofluoric acid
TFL	Total-etch feldspathic ceramic light-cured
TFD	Total-etch feldspathic ceramic dual-cured
TLL	Total-etch lithium disilicate ceramic light-cured
TLD	Total-etch lithium disilicate ceramic dual-cured
TAL	Total-etch alumina light-cured
TAD	Total-etch alumina dual-cured
SFL	Self-etch feldspathic ceramic light-cured
SFD	Self-etch feldspathic ceramic dual-cured
SLL	Self-etch lithium disilicate ceramic light-cured
SLD	Self-etch lithium disilicate ceramic dual-cured
SAL	Self-etch alumina light-cured
SAD	Self-etch alumina dual-cured
NFA	No-treatment feldspathic ceramic self-adhesive
NLA	No-treatment lithium disilicate ceramic self-adhesive
NAA	No-treatment alumina self-adhesive
F	Force
A	Cross-section area
AIC	Akaike information criterion
BIC	Bayesian information criterion
RMSE	Root mean square error
VIF	Variance inflation factor
SD	Standard deviation
Min	Minimum
Max	Maximum
